# Attention deficit hyperactivity disorder in prematurely born children: role of neuroinflammation caused by human cytomegalovirus infection

**DOI:** 10.1192/j.eurpsy.2025.1171

**Published:** 2025-08-26

**Authors:** B. Pavković, J. Skoric, M. Zaric, J. Lakic, M. Bozinovic Stanojevic, I. Paripovic

**Affiliations:** 1 Department of Mental Health; 2 Department of Pediatrics, Health Center “Dr Simo Milosevic”, Belgrade; 3 Specialist Practice for Child Psychiatry “Psiho Centar MM”, Nis; 4 Insitute of Neonatology, Belgrade, Serbia

## Abstract

**Introduction:**

Numerous studies have revealed the association between deficit hyperactivity disorder (ADHD) and brain inflammation due to immune system response to congenital or perinatal human cytomegalovirus (CMV) infection.

**Objectives:**

The aim of study was to examine the impact of neuroinflammation caused by CMV infection on the development of ADHD in prematurely born children.

**Methods:**

The medical records of 126 prematurely born children aged 7-11 were retrospectively analyzed. Participants were divided into two groups, the observed population of 56 children with ADHD and the control group without ADHD. Three parameters were observed, C-reactive protein (CRP) as an indicator of inflammation, IgM antibodies to CMV for etiological diagnosis of CMV infection and cranial ultrasound findings for the confirmation of structural changes in the brain.

**Results:**

Statistical analysis of our data showed the association between the onset of ADHD and the presence of congenital/perinatal CMV infection in prematurely born children (p<0.01). Nevertheless, these two variables had a very low positive correlation (phi coefficient 0.07173). The results did not show the association between elevated levels of CRP and presence of ADHD in prematurely born children (p>0.01), which confirmed that not every inflammation, regardless of the cause, was associated with ADHD. The analysis also confirmed the positive correlation between the variables listed in pairs: elevated levels of CRP and positive IgM on CMV, elevated levels of CRP and altered ultrasound neuroimaging findings, as well as positive IgM on CMV and altered ultrasound neuroimaging findings. All of these correlations speak in favor of the CMV caused neuroinflammation as etiopathogenetic basis in ADHD.

**Conclusions:**

In our sample CMV-induced neuroinflammation was associated with the development of ADHD in prematurely born children.

**Disclosure of Interest:**

None Declared

